# Ecology of the Western Queen Butterfly *Danaus gilippus thersippus* (Lepidoptera: Nymphalidae) in the Mojave and Sonoran Deserts

**DOI:** 10.3390/insects11050315

**Published:** 2020-05-19

**Authors:** Leslie Saul-Gershenz, Steven M. Grodsky, Rebecca R. Hernandez

**Affiliations:** 1 Wild Energy Initiative, John Muir Institute of the Environment, University of California, Davis, 1 Shields Ave., Davis, CA 95616, USA; smgrodsky@ucdavis.edu (S.M.G.); rrhernandez@ucdavis.edu (R.R.H.); 2 Department of Entomology and Nematology, 1 Shields Ave., University of California, Davis, Davis, CA 95616, USA; 3 USDA-ARS, Invasive Species and Pollinator Health Research Unit, 3026 Bee Biology Rd, Davis, CA 95616, USA; 4 Department of Land, Air, and Water Resources, 1 Shields Ave., University of California, Davis, Davis, CA 95616, USA

**Keywords:** citizen science, *Danaus gilippus thersippus*, iNaturalist, queen butterfly, milkweed, *Asclepias*, pyrrolizidine alkaloids, Mojave Desert, monarch butterfly, Sonoran Desert

## Abstract

The purpose of this study was to assess the ecological knowledge surrounding the western queen butterfly, *Danaus gilippus thersippus* (H. Bates). Specifically, our objectives were to synthesize existing data and knowledge on the ecology of the queen and use results of this assessment to inform the direction of future research on this understudied species. We identified six core areas for assessment: distribution, the biodiversity of plant resources, western queen and their host plant phenology, chemical ecology, and four key life history traits. We mapped the distribution of *D. g. thersippus* from museum specimen records, citizen science (e.g., iNaturalist) and image sharing app-based observations, along with other observational data enumerating all current known plant resources and long-range movements. We assembled 14 larval food plants, six pyrrolizidine alkaloids plants and six nectar plants distributed in the western Mojave and Sonoran Desert regions of the United States and Baja California. We report on its phenology and its long-range movement. Butterfly species have declined across the western US, and western monarch populations have declined by 97%. *Danaus g. thersippus* has received little research attention compared with its famous congener *D. plexippus* L. *Danaus g. thersippus’* desert distribution may be at its temperature limits for the species distribution and for its rare host plant *Asclepias nyctaginifolia.*

## 1. Introduction

Both rare and common butterfly species in California, United States (US), have declined based on a 35-year dataset of butterfly species due to the compounded effects of habitat alteration [1], increased use of neonicotinoid insecticides [2], and climate change [3,4,5]. Aridland butterflies may be even more vulnerable as such species and their host plants are often living at the upper limit of their physiological temperature tolerance [6,7]. Thus, aridland butterfly populations near agricultural areas are at risk from all four unique types of risks.

Plants are essential to the survival of butterflies. *Danaus gilippus thersippus* require host plants, which support their growth and larvae (such as milkweeds), as well as nectar plants to satisfy their caloric needs as adults. These plant resources have declined due to land development, agricultural intensification [8], herbicide resistant crop production [9,10], and changes in bee pollinator communities [11]. At least 104 species of native bees use milkweed species [12]. Native bee species that pollinate *Danaus gilippus* resource plants have declined, and their decline is also linked to an increased use of neonicotinoids [13,14], habitat alteration, and increased stress from pathogens [15]. The urgency to decarbonize US power plants has increased pressure on public lands to develop ground-mounted, utility-scale solar energy in the Mojave and Sonoran Deserts (California, US). Solar energy development in these natural environments has increased the rate and magnitude of habitat alteration in aridlands, which are hotspots of biodiversity [16]. In California, *D. g. thersippus* and desert populations of milkweed species co-occur, including the endangered milkweed, *Asclepias nyctaginifolia* [17]. Current studies estimate that approximately 80% of temperate-zone angiosperms depend on animals, mostly insects and mostly bees, to pollinate flowering plants [18]. In the western United States, most rare and threatened plants fit this finding as well [19].

*Monarch versus queen research productivity*. After decades of intensive research, focused on monarchs, scientists estimate a 97% decline in western monarchs *D. plexippus* abundance from its historic levels in the 1980s [20], which are congeners of *D. g. thersippus*. A scientometric “topic search” in the Core Collection in Web of Science (WOS), a non-public collection of databases and indexing service, for publications on queen butterflies using the general term “*Danaus gilippus*” yields only 50 scientific references for *Danaus gilippus*, most of which are studies of the eastern subspecies *D. g. berenice.* A search in WOS for publications on its congener, the monarch, using the term “*Danaus plexippus*” yields 728 references, demonstrating that western *D. g. thersippus* have received a fraction of the scientific attention that *Danaus plexippus* have received.

*Summary of Queen Life History Knowledge*. *Danaus g. thersippus*, like *D. plexippus*, require larval host plants with cardiac glycosides (CG). Like *D. plexippus*, *D. gilippus* sequester alkaloids for defense and are themselves insensitive to the effects of alkaloids, albeit through different molecular mechanisms [21]. *Danaus g. thersippus* males, like *D. plexippus* males also require nectar plants with pyrrolizidine alkaloids (PAs) to attract females during courtship and mating [22]. *Danaus g. thersippus* are key herbivores in regions where habitat alteration from ground-mounted, utility-scale solar development, housing development, and agriculture expansion exert pressure on their complex ecosystems. *Danaus g. thersippus* occupy a similar niche as *D. plexippus*, but with some important differences. *Danaus g. thersippus* are commonly found in the Mojave and Sonoran Deserts and may serve as additional models of migratory behavior in the western aridlands of the US.

*Purpose and Objectives*. The purpose of this study is to conduct an ecological assessment of knowledge of *D. g. thersippus* that synthesizes existing data and knowledge on the ecology of the queen. Our objective is to use results of this assessment to inform the direction of future research and springboard future research on this understudied species. We identified six core areas for assessment: distribution, the biodiversity of plant resources, phenology of these plants and of *D. g. thersippus*, its chemical ecology, and life history traits (i.e., movement patterns; diet; mating behavior; and predators, parasites and pathogens). Specifically, our objectives were to (1) map the distribution of *D. g. thersippus* using all known and available geodata including, museum specimen records, citizen science and image app-based observations, along with other observational data; (2) identify and compare larval host, pyrrolizidine alkaloids host plants and nutritive nectar plant resources and determine their phenology, (3) synthesize phenological data of *D*. *g. thersippus*, (4), synthesize phenological data of the three categories of host plants, (5) summarize chemical ecology, and, (6) assess four key life history traits including long-distance movement behavior.

This research is part of a project to explore the impact of ground-mounted utility-scale solar development on wildlife in the western deserts of the United States [23]. The urgency to decarbonize US power plants has increased pressure on public lands which support wildlife and which are adjacent to protected areas to develop ground-mounted, utility-scale solar renewable energy in the Mojave and Sonoran Deserts. This has increased the rate and magnitude of habitat alteration in these ecosystems, which are hotspots of biodiversity [16]. Aridlands are complex crosslinked networks of soil organisms, primary producers, pollinators, herbivores, predators, parasites and microbiota, with highly variable abiotic resources, including multi-year drought cycles. Specifically, the Ivanpah Solar Electric Generating System (ISEGS) in the northern Mojave Desert is adjacent to a rare population of the milkweed, *Asclepias nyctaginifolia* A. Gray [17], which is a larval food plant of *D. g. thersippus* [24]. Several other solar facilities east of Joshua Tree National Park border populations of other milkweed and other nectar species used by *D. g. thersippus*. Thus, the purpose of this paper is to assess and synthesize the current literature to determine the distribution, phenology, host plant relationships, and movements of this desert butterfly *D. g. thersippus*, which is interwoven in the complex ecosystems of the Mojave and Sonoran Deserts.

## 2. Materials and Methods

### 2.1. Distribution and Phenology

**Figure 1:**
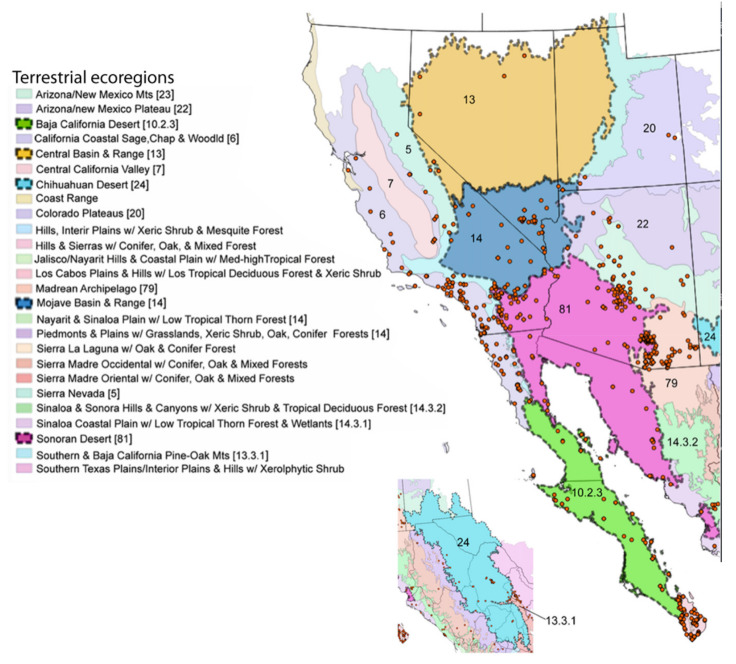
Distribution of *Danaus gilippus thersippus* in western United States and western Mexico. The 1008 data records were sourced from 348 museum specimen and 660 observation records [28,35] highlighting Mojave, Sonoran and Great Basin Desert ecoregions in Arizona, California, Nevada, Utah and Mexico but including all areas of these states (map adapted from Level III US Environmental Protection Agency (EPA) map data [37]). The numbers on the map correspond to the EPA ecoregions of focus where *D. g. thersippus* occurred. Inset map shows observations near and within the Chihuahuan Desert ecoregion.

**Figure 2:**
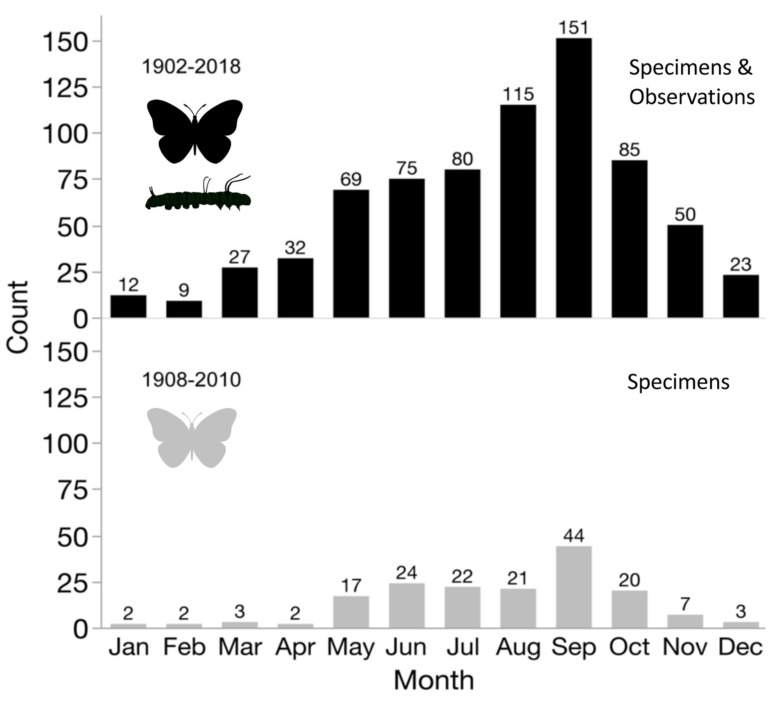
Phenology of *Danaus gilippus thersippus* across their range in Arizona, California, Nevada, and Utah. This phenological analysis is based on a museum specimen records (below, *N* = 167) and museum specimen and citizen science and image sharing app-based observation records combined (above, *N* = 548) [28] (Supplementary Tables S1 and S2). Specimen records are comprised of adult butterfly stage data (100%). Combining museum specimen records with citizen science and image sharing app-based observational data increases numerical strength for analysis; however, observational data from multiple sources often lacked life stage designation.

Locality and phenological records for *D. g. thersippus* in western United States and Mexico were assembled from databases on the Global Biodiversity Information Facility (GBIF) and Biodiversity Information Serving Our Nation (BISON) (Supplementary Table S1) and Computarización y actualización de la curación de la Colección de Lepidóptera del Museo de Zoología “Alfonso L. Herrera” y su base de datos MARIPOSA, FASE I, which includes museum specimens and citizen science observational records from iNaturalist, and the Lepidopterists’ Society Season Summary website for *D. g. thersippus* hosted by the Florida Museum of Natural History at the University of Florida [25]. Data from “unknown” sources on these sites were redacted and not used. Filtered locality data with duplicates removed for the western US states of Arizona, California, Nevada and Utah were used to produce the *D. g. thersippus* distribution map (Figure 1). Phenological data sources include peer-reviewed literature and images of plant associations from collections, iNaturalist, and Flickr after plant identifications were confirmed by James M. Andre, Director of the Granite Mountains Desert Research Center, University of California, Natural Reserve System. We sorted the data by locality and date of collection, removed locality duplicates from the same date, and produced a count of dates in each month when *D. gilippus* were collected from each unique locality to produce the distribution map [25,26] (Figure 1 and Figure 2). This data is summarized in Table 1. Specimen records in museum databases are assumed to be adults and correlate with a specimen, while observation records are a mix of adults and larval stage and occasionally egg stage data records. However, these are difficult to distinguish in iNaturalist, Flickr, BugGuide and other online databases because the life stage has not been designated and would require viewing each individual record and making a corresponding database of life stage for each record with their identification number. The addition of life stage designation data to online databases would increase the scientific value of citizen science and image sharing app-based Lepidopteran databases, thereby increasing the number of records available for analysis.

**Figure 3:**
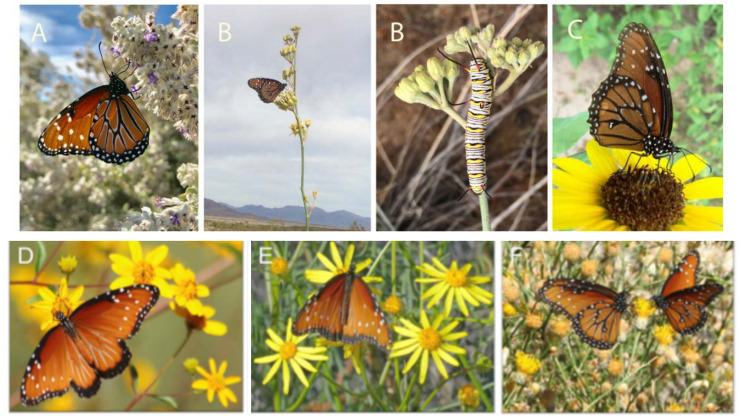
(**A**) *Danaus gilippus thersippus* nectaring on *Condea emoryi* 15 March 2019, in a wash northwest of Desert Sunlight Solar (© N. Gershenz and L. Saul-Gershenz all rights reserved); (**B**) *Danaus gilippus thersippus* nectaring on *Asclepias subulata* on 15 April 2019 and caterpillar feeding on *A. subulata* 5 May 2019 in wash northwest of Desert Sunlight Solar in 2019 (© L. Saul-Gershenz all rights reserved); (**C**) *Danaus gilippus thersippus* nectaring on *Helianthus annus,* 30 mi south of Animas, Hidalgo Co. NM, 27Aug 2008 (J. S. Ascher © all rights reserved); (**D**) *Danaus gilippus thersippus* in Madera Canyon, Santa Cruz Co., AZ, 9 Oct. 2014 on *Heliomeris longifolia* [57]; (**E**) *Danaus gilippus thersippus* on *Xanthisma spinulosum* [57] in Pima Co., AZ, 28 Nov. 2018; (**F**) *Danaus g. thersippus* in San Carlos, Mexico, 8 Nov 2011(© Carol H. all rights reserved) on *Acamptoppaus sphaeorcephalus* [57]. Images D, E, F [accessed on Flickr, 30 Oct 2019].

We collated the phenological data of *D. g. thersippus*, and, to avoid collection bias, we counted only unique locality/month/day records for all years. Therefore, if two specimens were collected at one site on the same date, we considered it one record. We visualized the phenological data for *Danaus g. thersippus* (Figure 2). We assessed the phenological data for all plant species recorded, based on peer-reviewed literature, databases (Supplementary Table S1), photographic documentation (for example Figure 3, Supplementary Figure S1), that were documented to be used by queens. We predicted that plant species used by conspecifics were used by other *Danaus* species if they were sympatric with *D. g. thersippus* and if they contained appropriate larval or nectar chemistry such as pyrrolizidine alkaloids (Table 2, Table 3 and Table 4). If use was documented, we cited the reference. We then calculated the average plant resource availability per month (Supplementary Figure S2).

### 2.2. Host Plant Use and Phenology

We searched citizen science and image sharing app-based sites BugGuide, Flickr, and iNaturalist to find temporal observations of host plant use. These data on these sites were then confirmed by cross-checking host plant range data on Calflora and DiscoverLife in addition to botanical authorities.

## 3. Results

### 3.1. Distribution and Phenology

We located 720 data points, (172 specimen records, 548 observation records) in the US from 1930 to 2018 and used these to construct the distribution map [27] (Table 1, Figure 1). Thus, 24.89% of the data come from museum specimen records and 76.11% come from observation records via iNaturalist (*N* = 417), BugGuide (*N* = 45) and Butterflies and Moths of North America (*N* = 82) and other sources (*N* = 4). We located 270 distribution data points (after 71 duplicate data were redacted) based on a total of 166 museum specimen records and 104 observation records in Mexico (Table 1). The specimens were collected from 1935 through 2019 with a peak of specimen records in 1961 of 39 specimens. These were collated from 26 institutions [28] (SI-1). *Danaus g. thersippus* are found throughout the Mojave Desert in CA: Imperial Co. Inyo Co., San Bernadino Co., Riverside, Co., San Diego Co.; NV: Clark Co. and they are common throughout the Colorado Desert in California, the Sonoran Desert in AZ [29,30] (Figure 1, Table 2, Table 3 and Table 4). Their distribution extends south to northwestern Mexico [31,32]. This species occurs in the Chihauhuan desert as well but this was beyond the scope of this study. Records assembled for Figure 1 show a distribution in the Mojave and Sonoran Deserts (ecoregions 14 and 81) and the California coastal range (ecoregion 6) east into the Arizona/New Mexico Mountains (ecoregion 23) and Madrean Archipelago or sky islands (ecoregion 79) and south into Baja California (ecoregion 10.2.3), Sinaloa and the Chihuahuan Desert in Mexico (ecoregions 24, 14.3.2, 13.3.1, and 9.6.1, respectively). Current records show that the California central valley (ecoregion 7) and the Great Basin Desert (ecoregion 13) lack *D. g. thersippus,* which is likely due to different abiotic and biotic processes.

According to Coolidge [33] and Emmel and Emmel [29], adults are found along the Pacific coast to San Diego and in the coastal mountains during fall; however, they are less common in the coastal region during spring and summer and are more typically found in the desert regions [34]. Late summer and fall records from the coast most likely represent adults that have dispersed from the desert [35]. Brown [35] suggests that there may be small stable coastal populations in San Diego County (Co.) or elsewhere where larval host plants are present. Some consider the San Joaquin Desert to possess similar climatic conditions and habit in Kern Co. and Fresno Co. and classify these areas as desert [36], hence we have indicated plant locations in Kern Co. as well (Table 2, Table 3 and Table 4).

Understanding the complex phenology all life stages of *D. g. thersippus* is a critical element of the queen’s ecology and requires an understanding of its behavior patterns in the western deserts. Currently, we have observations from different studies documenting its presence from January to December in different life stages and a flight period from April to November [29]. Specimen records from Arizona in January as well as in Orange Co. [34] indicate they are active in fall months but also active in summer months (Figure 2, Supplementary Table S2). Records from the western states of Mexico (Baja, Sonora and Sinaloa) suggest that queens are active in all months but most active in October and November. More adult and larval records are needed to complete a full picture of this species’ phenology across its entire geographic range in the western US and Mexico. While the volume of scientific collecting has declined, the observation recording has increased. However, some of these records lack coordinate data, host plant associations and life stage designation in the database and other behavioral data (nectaring, mating, ovipositing, larval feeding, etc.), which would make these observations more useful scientifically.

### 3.2. Movement Patterns

Migration is defined as “an adaptation to resources that fluctuate spatiotemporally either seasonally or less predictably” [38]. Dingle and Drake [38] also noted that it often occurs preemptively before resources disappear. *Danaus g. thersippus,* in the desert in particular, must adapt to ephemeral nectar, larval and pyrrolizidine alkaloid (PA) host plant resources, with brief availability windows, and drought cycles that affect the diversity and abundance of host plants, with preemptive strategies before food sources decline [38,39]. Triggers for seasonal responses such as photoperiod or endogenous rhythms, changes in humidity, or food availability may cue movement patterns [39] of *D. g. thersippus*. However, *D. g. thersippus* have not received the intensive research tagging efforts that have advanced the understanding of *D. plexippus* migration behavior. Close inspection of museum specimen labels and published observations from other web-based sources such as GBIF, BugGuide, DiscoverLife, Scan, and iNaturalist provide some nascent evidence of seasonal movement (Supplementary Table S2). Tagged adults document movement of three different individuals of *D. g. thersippus* moving west from Arizona to California (598.85 km, 671.42 km, and 1404.31 km) (*N* = 3, Supplementary Table S2), suggesting that this species is capable of moving long distances. However, we were only able to locate three tagged individuals during our searches. If these are part of a systematic tagging effort, it would be more informative. Others individuals remain active and appear to remain in desert habitats in winter (Supplementary Table S2). Scott [40] states that *D. g. thersippus* fly from April to Nov in southern California and southern Nevada. This is an area that merits more research for this species.

### 3.3. Diet

*Danaus g. thersippus* require three major dietary categories for survival: larval host plants, adult nectar plants for nutrients and energy and nectar plants that contain pyrrolizidine alkaloids (PAs) required for courtship and mating. They use different plant species across their geographic range in the western Mojave and Sonoran Deserts and temporally throughout their annual lifespan (Table 2, Table 3 and Table 4, Supplementary Figure S2). The nectar of 90% of most species of plants has amino acids [41], 50% has detectable lipids, 60% has phenols [42,43] in addition to vitamins, minerals [44,45], alkaloids which have antimicrobial affects, terpenoids [44,46,47,48], and phytosteroids [44]. In addition, some nectars contain antimicrobials and antioxidants ([41] and references within). The availability of nectar can be affected by fluctuations in soil moisture resulting from drought [49], which is an important factor in arid landscapes. Nectar sources contain other constituents including antioxidents [50], alkaloids, microbiota such as bacteria, yeast, and fungi, which may serve functional roles such as pathogen control. Infected insects may employ antimicrobial phytochemicals against their own diseases [51]. Singer et al. [51,52] showed that *Apantesis* (formerly *Grammia*) *incorrupta* (Hy. Edwards) (Lepidoptera: Erebidae: Subfamily Arctiinae) larva ingestion of PA plant toxins improve the survival of parasitized caterpillars by conferring resistance against *Exorista mella* (Walker) flies in the family Tachinidae, compared to infected caterpillars on feeding on plants without PAs. The alkaloid gelsimine, which is found in the nectar of *Gelsemium sempervirens*, when fed on by *Bombus impatiens*, was found to reduce the gut protozoan *Crithida bombi* [53] in lab experiments. Small amounts of leaf herbivory by *D. plexippus* caterpillars, consistently resulted in induced increases in foliar cardenolides of *A. syriaca* [54] and research by Vannette and Hunter [55] suggest that mutualistic partners such as *Scutellospora pellucida* fungi mediate latex and cardenolide production in some species of *Asclepias*. Some amino acids in nectar have the potential to modify insect behavior by stimulating insect chemosensory receptors ([41], review).

The nectar source desert lavender *Condea emoryi* (Torr.) Harley and J.F.B. Pastore contains secondary compounds such as butelinic acid which have antimicrobial, antitumor, and anti-inflammatory properties [56] and are important resources for *D. g. thersippus* and other desert butterflies (Table 3, Figure 3A, LSG field observations 2018–2019). *Condea* shrublands appear to be limited by temperature, as it does not occur > 700 m in the desert mountains and it does not occur very far north into the Mojave Desert [57]. *Condea emoryi* are found in low washes and tolerate a high degree of flood disturbance that occurs during high precipitation years which occur about every 10 years during winter and monsoon periods in summer. It is a long-lived species that re-sprouts following floods. Populations occur north and east of Joshua Tree Nat. Park (Table 3, Figure 3). 

Several sources cite the use of desert palafox *Palafoxia arida* B.L. Turner as an important nectar source of *D. g. thersippus* including Pfeiler et al. (Table 5 in [32]). It is an annual which is locally abundant on dunes and sandy washes of bajadas appearing in April through May in Mojave dunes throughout California and northern Mexico [57]. As noted in Table 2, Table 3 and Table 4, *D. g. thersippus* caterpillars specialize on plants in the genus *Asclepias*, and the related genus *Funestrum* while adult butterflies collect nectar from plants in many families (Asteraceae, Boraginaceae, Fabaceae, Lamiaceae, Apocynaceae). We used data from citizen science and image sharing-based data such iNaturalist, BugGuide, and Flickr to expand the dietary knowledge of *D. g. thersippus* (Table 2, Table 3 and Table 4).

Adult female butterflies require nectar with sugars, amino acids and other nutrients for egg production and oviposition (Figure 3). Both males and females feed on plants containing pyrrolizidine alkaloids (PAs). However, males require PAs to produce their courtship attractant to mate with females [see chemical ecology below). All *Senecio* species tested (186 spp.) contain PAs [58], suggesting that *Senecio faccidus* var. *douglasii* and *Senecio faccidus var. monoensis* (Table 3) are sources of PAs in the Mojave Desert where they occur. PAs were found in all plant tissues of *Senecio*; however, the inflorescences contained 90% of the total PAs [103,104]. PAs have been found in the desert species *Amsinkia tesellata* [84,105] and sixteen species of *Eupatorium* and one species of *Ageratum* (Table 3). *Amsinkia tessellata, Senecio* and *Cryptantha* are all present in the Ivanpah Valley region of the Mojave where *D. g. thersippus* occur (Figure 1). *Tournefortia* bait experiments of ([61], Table 2) list species found in the western US which attracted male *D. gilippus berenice*. In Florida, *D. gilippus* use some of the same food plants as *D. plexippus* [61], which suggests that other *Danaus* subspecies may also share host plants with *D. plexippus* in other regions of the US. Nine species of *Cryptantha* have been shown to contain PAs [84] and several species of *Cryptantha* occur broadly in the western deserts and thus may be a source of PAs for *D. g. thersippus*. However, desert species of *Amsinkia*, *Cryptantha,* and *Senecio* require field experiments and observations to provide confirmation of their use and a deeper understanding of the nexus between *D. g. thersippus* butterflies, its host plants and local populations across aridlands throughout the western US and Mexico.

Analysis of the plant species resource availability across the entire Mojave and Sonoran ecosystem using the phenology of *D. g. thersippus* larval and adult nectar plants including PA sources in the western US deserts yielded a preliminary projected food plant availability in this ecoregion (Supplementary Figure S2). The highest average food plant resource availability occurs in the months of May 0.43, April 0.7, May 0.83, June 0.83, July 0.66, August 0.53, and September 0.51, October 0.4 (Supplementary Figure S2), which corresponds with *D. g. thersippus* peak butterfly-larval phenological presence analysis in Figure 2.

### 3.4. Mating Behavior

*Danaus g. thersippus* eclose shortly after sunrise (30 min. past) [62]. Male *D. g. thersippus* patrol all day [62]. Males release an attraction pheromone during courtship that functions as a premating isolating mechanism [40,105]. The duration of copulation is 100 min to 12 h [29,40,106] or 4.5 h and longer according to Pliske and Eisner [107]. *Danaus gilippus* fly in copula [30] and the male usually carries the female *D. gilippus*: [40,108]. Females can mate up to 10 times [40]. Unreceptive females simply fly away [40]. *Danaus gilippus* males use PAs in courtship as honest advertisements of nuptial gifts of protective PAs which they transfer to females upon mating; females then transfer these PAs to the eggs [22]. Thus, plants containing PAs are as essential to the survival of *Danaus* butterflies as are milkweed plants with defensive cardenolides.

### 3.5. Chemical Ecology

The variability of cardenolide chemistry in host plants may affect the survivorship of larvae and adult butterflies. The choice of host plant oviposition site by females is one the most importance choices to ensure offspring success after mate choice. Due to the co-evolution of *Danaus* and the chemical ecology of its host plants this is a fertile subject of research with a growing body of literature.

*Danaus g. thersippus* in the western deserts rely on three categories of plants resources without which they cannot survive. The specialized host plants that contain cardenolides in the genera *Asclepias* and *Funastrum* (Apocynaceae) and other secondary compounds are required by the larva. The plants used by males to collect the precursor of their sex attractant are essential for attracting females, courtship and mating. In addition, both females and males require nectar sources for their entire adult lifespan which contain an appropriate suite of nutrients and energy resources. If one of these categories of host plant resources is absent, *D. g. thersippus* and *Danaus* populations in general will not be sustainable. *Danaus g. thersippus* are mobile flyers so they may mate in one location and oviposit their eggs in a different location; however, nectar resources are required for fueling travel and searching behavior.

Plants containing pyrrolizidine alkaloids possess two types of volatile compounds that attract male butterflies. Substances on the plant surface act as phagostimulants when a butterfly lands on a plant. Male *D. gilippus* sequester the precursor for the production of dihydropyrrolizines from plants (Table 3) that they release via their hairpencils and that contain pyrrolizidine alkaloids [107,109] review. This attractant called danaidone attracts females to males during courtship [110]. Plants in the genus *Senecio*, (Figure S1) *Amsinkia*, *Ageratina* and *Cryptantha*, contain pyrrolizidine alkaloids sequestered by adult males from plants, which is transferred to females during mating, and then transferred by the female to the eggs [22]. It has been suggested that the eggs are protected from predators and parasites as a result but this requires further testing.

Milkweed plants in the genus *Asclepias* produce up to 200 structurally different cardenolides, which appear to all have the same inhibition of the Na^+^/K^+^ ATPase in animal cells [69]. These cardiac glycosides block a key transmembrane carrier in animals [111]. Cardenolides are steroids which occur as glycosides with one or more sugars attached in plant or insect tissue [112]. For a detailed discussion of the chemical structure of cardiac glycosides, see Malcolm [112], and, for a thorough review of all aspects of the chemical ecology of cardenolides and danaids, see Agrawal et al. [21]. The polarity of cardenolides determines its absorption rate in the herbivore [21]. Cardenolides can be found in all *Asclepias* species [21,109], and all plant tissues, including latex, and nectar and cardenolide expression is tissue specific [21,112,113]. In addition, the concentration varies in time and in type of cardenolide, which vary in polarity [112,114,115], and in the diversity of compounds [116]. The binding of cardenolides to Na^+^/K^+^ ATPase is temperature dependent [21]. Latex has higher concentration of cardenolides in some species such as *A. eriocarpa* [21,115,117]. Moranz and Brower [118] also found that concentrations of cardenolides, which varied temporally in *Danaus gilippus*, were mediated by their host plants.

Environmental variability can have significant impacts on foliar traits, such as soil moisture content [49], and seasonal temperature variation, which will have an impact on the concentration of toxins experienced by herbivores [113]. Secondary metabolism is influenced by water stress, which is relevant in desert systems and CO_2_ level; a 20%–30% decline in cardenolides was recorded from water stress (86% reduction in leaf H_2_O potential). Cardenolide content increased with elevated CO_2_ (1000 ppm) [21,119]. Herbivore damage can induce cardenolide expression as well [72,120,121]. The genus *Asclepias* contains cardenolides and other alkaloids (Table 2) [21] and variation within a species is a heritable trait [80]. Paired comparison field experiments of plants in full sun or deep shading resulted in decreased concentration and diversity of cardenolides [21]. Cardenolides may function defensively against bacteria, fungi, protozoa and viruses [122,123,124,125]; however, research has yielded varied results. Vannette and Hunter [55] found a root infection by fungal species *Scutellospora pellucida* which induced increased leaf cardenolides and plant growth in *A. syriaca* but found no effect with fungus *Glomus etunicatum*.

The ability to store cardenolides is different for different species of milkweed species depending on the polarity of the cardenolide glycosides. Less polar cardenolides are more able to cross membranes [121,126,127]. In addition, female herbivores store a higher rate of toxins than males when reared on plants of equal concentration [126]. In addition, cardenolides such as calotropin and calactin can be found at different ratios in host plants and selectively concentrated differently in *D. g. thersippus* and *D. plexippus* [128]. *Danaus gilippus* stored calotropin, 10–12 times less than *D. plexippus* but not calactin when both were fed on *Asclepias curassavica* [87]. These two compounds, which are present in *A. curassavica* and other species are very effective in inducing animal emesis [88]. In a study by Oyeyele and Zalucki [129], sister species *D. plexippus* laid 70% of their eggs on low foliar cardenolide plants, concurring with another study with *Asclepias curassavica* in which butterflies preferred plants with lower cardenolides as well [90]. Species having higher concentrations of total cardenolides tend to have fewer polar compounds on average, thus having a smaller number of polar cardenolides overall [113].

Researchers have hypothesized that more toxic species not only have high concentrations of cardenolides, but also more apolar forms, which are more easily absorbed in the insect hemolymph [21,113]. Both *D. g. thersippus* and *D. plexippus* are cardiac glycoside insensitive; however, *D. gilippus* have a different molecular modification at position 122 than *D. plexippus* [130].

Agrawal et al. [131] documented latex exuded in response to leaf damage in 53 species of *Asclepias*, including some found in the western deserts. Latex response was high in *Asclepias erosa* compared with *A. subulata, A. albicans, A. nyctaginifolia, A. fascularis, A. asperula, A. speciosa, A. vestita, A. latifolia, A. linaria*, respectively (16.059, vs. 0.228, 0.234, 0.461, 0.457, 0.840, 0.819, 4.766, 5.925, 5.991; Supplementary Table S2) [131]. This same pattern was not repeated in cardenolide content [92]. Latex-transporting canals are pressurized so when a leaf or vein is cut, latex flows and coagulates rapidly, drying into a glue-like substance. Hence, it is considered a plant defense [93]. Young *D. gilippus* caterpillars vein cut to block the flow of latex to feeding sites to counteract this defensive secretion of milkweeds [132]. “Self-medication” has been attributed to the behavior of parasite-infected *D. plexippus,* which lay their eggs on antiparasitic milkweed to protect their larval offspring from parasitic growth when they hatch [124].

### 3.6. Predators, Parasites and Pathogens

Tachinid flies *Lespesia archippivora* Riley [Diptera: Tachinidae) [133] attack the larva of *D. g. thersippus* [134]. *Danaus gilippus* is parasitized by two chalcid wasps, *B. ovata* Say [134], *Brachymeria annulata* Fabricus [Hymenoptera Chalcididae) [135]. *Ophryocystis elektroscirrha* is a protozoan parasite that was first recovered from *Danaus plexippus* and *Danaus gilippus* butterflies in Florida in 1966 [136]. New infections occur when larvae ingest parasite spores as they feed on contaminated egg shells or milkweed leaves. Most spores are transmitted from infected adults to their offspring (vertical transmission), although horizontal transmission may also occur. Following ingestion, spores lyse in larval guts. Heavily infected adults have difficulty emerging from their pupal cases and expanding their wings, although adults with low parasite loads appear normal [136]. McLaughlin and Myers [136] found an *Ophryocytis* infection rate of 22% and 50% on the scales of adult *Danaus g. berenice* butterflies in Florida. A major cause of mortality in reared *D. gilippus* is a highly contagious polyhedral virus [136]. Birds are well documented predators of *D. plexippus* [137] and likely prey on *Danaus g. thersippus* as well.

## 4. Discussion

### 4.1. Distribution

We assembled the spatial distribution *of D. g. thersippus* in the western USA and Mexico based on current literature, digital specimen records, observations, and image records (Figure 1). This distribution encompasses Arizona, California, Nevada, New Mexico, Utah, and Baja California to focus on the Mojave and Sonoran Desert distribution of *D. g. thersippus*. This analysis documents that *D. g. thersippus* uses both desert and coastal regions. It also suggests that the Great Basin Desert is not a preferred habitat perhaps due to climatic and other biotic limitations or this may be the result collecting-observation bias. We did not include the Chihuahuan Desert in this inquiry due to the current geographic focus of our research and field surveys; however, this species does extend into the Chihuahuan Desert as shown by museum and observation records [25,28]. Of note, is the absence of *D. g. thersippus* in the Central Valley likely due to anthropogenic activities including habitat alteration, resulting in the loss of all three categories of required host plants [8], and increased use of insecticides and herbicides [10,13,14]. We have also documented two populations of *D. g. thersippus* adjacent to protected areas: one in the interspace between the Ivanpah Solar Electric Generating System (ISEGS) and the Mojave National Preserve and a second near Desert Sunlight Solar and Joshua Tree National Park.

Analysis of the available distribution data reveals that only 24.89% of the data come from museum specimen records, revealing the shortage of this type of information for this species in the desert ecoregion. Observation records for adult and larval queen butterflies are easily identified; however, host plant associations would be best documented with specimen-based records so they can be verified by specialists.

### 4.2. Movement

Three tagging records document three specimens of *D. g. thersippus* long-distant movement events up to 1404 km from east to west. These appear to be in a manner similar to migratory movement [38] which its congener *D. plexippus* undertakes annually. All three movement records occurred in the fall (two in September and one in October), hence the time of year is also suggestive. The data provide some early hint that this population may move from east to west seasonally. However, the movement patterns and ecology of *D. g. thersippus* needs to be greatly expanded to understand the movement biology of this species. These three records all came from Lepidopterists’ Society Season Summary [25] website which highlights the benefit of exploring multiple forms of data and organizations involved in data aggregation and more structured, and coordinated research for this species.

### 4.3. Phenology

We assembled the phenology of *D. g. thersippus* across its entire western range in the US covering Arizona, California, Nevada, New Mexico, Utah, and Baja California. Our analysis of phenological data from museum records and citizen-based observations (Figure 2, Figure S2) show that June, July, August, September and October are the peak activity periods for *Danaus g. thersippus* butterflies and larvae across their range in western range. Detailed site-specific research is required across their range to provide a more accurate phenology that can separate larval from adult phenology and to reveal if this species has migratory patterns. Plant record databases and literature show that April through Aug are the highest average periods for host plant (larval, nectar and pyrrolizidine alkaloid plant) resource availability across the Mojave and Sonoran Deserts. However, more detailed local site-specific phenology for each plant species’ emergence and bloom period is needed to make this information biologically meaningful. Phenology can also change significantly with changes in precipitation and temperature annually, particularly in arid ecosystems where species are living at the upper limit of their physiological temperature tolerance [6,7].

### 4.4. Diet

The use of online photographic databases, such as BugGuide, iNaturalist, Flickr and those associated with DiscoverLife, provided documentation of host plant use, including the locality of host plant, and phenological data, including flowering or vegetation availability for the larval stage. Most images used were focused on the adult butterfly stage, due to the ease of identification. Plant species identification was sometimes challenging. Additional photographs with detailed close-ups of flower structures and leaf shapes in focus would improve the research quality of these images. Observations on all these digital databases that show multiple taxa would benefit by including latitude and longitude coordinates.

A great deal more research is needed on both the larval and adult diets of *D. g. thersippus* across the desert ranges. Researchers require details about which specific species of nectar plants are used to sustain this long-lived butterfly, temporally beginning with its emergence from its chrysalis, through courtship, mating, searching for oviposition sites, and spatially as plant resources change in different localities and over time as the seasons progress in the extreme temperatures of desert climates inter and intra-annually. More research is needed to understand how other organisms, including other herbivores [138] and humans, affect the resources required to sustain populations of these and other desert butterflies and pollinators of their host plants, which are mostly bees. Loss of larval and adult host PAs and nectar plants resources is one of the major factors in the 97% population decline of *D. plexippus* butterflies in the western United States along with insecticide use [20], Pelton). *Danaus gilippus thersippus* is equally vulnerable and of interest scientifically due to its behavior, chemical ecology and hypothesized long-distance movement, which is similar to the monarch butterfly. In addition, *D. g. thersippus’* host plant species may be at their temperature limits due to its distribution in desert ecoregions, hence alterations in the regional hydrology or climate might adversely affect its ecological network as well.

### 4.5. Chemical Ecology

The chemical ecology of *D. g. thersippus* closely resembles the chemical ecology of *D*. *plexippus*, which has been thoroughly and eloquently reviewed by Agrawal et al. [21]. Of note is the paired comparison field experiments of plants in full sun or deep shading, which resulted in a decreased concentration and diversity of cardenolides [21]. The impact on plants containing cardenolides merits further assessment where the distribution of *Asclepias* species, *Danaus* species and ground-mounted utility-scale photovoltaic and concentrated solar energy (USSE) development intersect [139]. The shading of soil and host plants used by *Danaus* species may have ecological consequences on this complex network across both spatial and temporal scales. The cardenolide and pyrrolizidine alkaloid concentration of *Danaus* host plants is critical to the future of this and related species.

### 4.6. Predators, Parasites and Pathogens

*Danaus g*. *thersippus* is parasitized by tachinid flies *Lespesia archippivora* [133], and by two chalcid wasps, *B. ovata* [133] and *Brachymeria annulata* [135]. *Ophryocystis elektroscirrha* is a protozoan parasite that infects *D. g. thersippus*. Ophryocytis infection rate was 10% and 50% on the scales of adult *D*. *gilippus* from Florida in experiments [140,141]. A major cause of mortality in reared i. *gilippus* is a highly contagious polyhedral virus [136]. In addition, birds are well documented predators of *D. plexippus* [137] and likely prey on *D*. *g*. *thersippus* as well.

### 4.7. Suggestions for Future Studies

The purpose of this paper is to assemble the current information available on *Danaus g. thersippus* in the western Mojave and Sonoran Deserts of the US. This is particularly critical in light of the decline in numbers of all butterfly species due to rapid large-scale development in the western deserts from urban expansion, utility-scale ground-mounted solar development in and near protected landscapes, and the increased use of systemic insecticides. Our intent is to stimulate further research on *D. g. thersippus*, due to its similarities to and differences from its close congener *D. plexippus*, by giving a preliminary roadmap to future work. In addition, the intersection of *D. g. thersippus*, their chemical ecology, complex relationship with their host plants, their movement ecology and vulnerability due to their distribution in Mojave and Sonoran Deserts and coastal California merits research attention.

## 5. Conclusions

We assembled the distribution of the queen butterfly *Danaus gilippus thersippus* in the western U.S. states of Arizona, California, Nevada and Utah and western Mexico based on 1008 data points, (182 specimen records, 556 observation records) in the US and 166 museum specimen records and 104 observation records in Mexico to construct its distribution map [27] (Figure 1).

We assembled the phenology of *D. g. thersippus* in this ecoregion using data based on a combination of museum specimen records (24.8%, *N* = 184) and observation records (75.1%, *N* = 556).

We assembled a referenced table of *D. g. thersippus’* dietary resources in the western deserts, including 14 larval host plant species records, six pyrrolizidine alkaloid host plant records, and six nectar host plant records with focus on the Mojave Desert and Sonora Desert ecoregions in the western US and Mexico. This is a starter list to stimulate much needed additional research.

We report on the long-distance movement behavior of *D. g. thersippus* moving from east to west, data assembled from the Lepidopterists’ Society Season Summary [25].

We assembled a phenology of larval and adult host plant resource availability by month in the Mojave and Sonoran Deserts (Figure S2).

We summarized the mating behavior, chemical ecology, predator, parasites and pathogens attacking queen butterflies focusing on *D. g. thersippus* in the western United States.

## Figures and Tables

**Table 1:** Summary of data analyzed to determine *Danaus g. thersippus* distribution in deserts and phenology.

Location	Specimen Based Records	Observation Based Records	Total Data Records
US	182	556	738
Baja Ca	53	13	66
Baja Ca Sur	96	15	111
Sonora	12	9	21
Sinaloa	5	67	72
Western Mex. Subtotal	166	104	270
**Total**	**348**	**660**	**1008**

**Table 2:** *Danaus gilippus thersippus* larval host plant resource availability. *Danaus g. thersippus* plant resource availability in the western desert regions of the Mojave and Sonoran Deserts in the US and Mexico, including larval host plants, nectar plants and pyrrolizidine alkaloid plants used by adult *D. g. thersippus*.

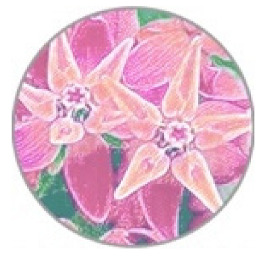 Host plant used by queen butterflies	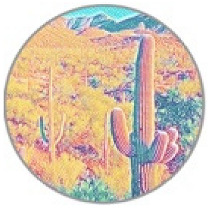 Found in desert	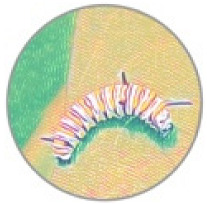 Larval host plant	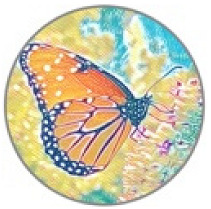 Nectar plant	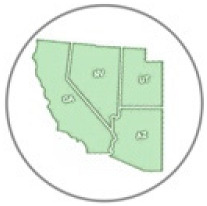 Desert distribution in Arizona (AZ), California (CA), Nevada (NV), Utah (UT) and Mexico (Mex)
**Apocynaceae**				
***Asclepias albicans*** S. Watson	Y [58,59]	Y [29,59,60]	Y [58]	AZ: La Paz, Maricopa, Pinal, Yuma [29,61] CA: Imperial, Riverside, San Bernardino, San Diego; Mex: Baja, CA
*Asclepias angustifolia* (Schweigg)		Y [62]		AZ: Cochise, Pima, Santa Cruz; Mex [58,61]
*Asclepias asperula* (Decne.) Woodson		Y [58,60]		AZ: all counties except Yuma [29,61]; CA: Riverside, San Bernardino; NV [63]; Mex
*Asclepias curassavica*		Y [29,40]		AZ: Pima; CA: Los Angeles, Orange, San Diego [29,64]
*Asclepias cutleri* Woodson	Y			AZ: Apache, Coconino, Navajo; UT [58,61]
***Asclepias erosa*** **Torr.**	Y [61]	Y [29,58]		AZ: Coconino, La Paz, Mohave, Yuma [61,65]; CA: Imperial, Inyo, Riverside, San Bernardino, San Diego [66]; NV; Mex: Baja CA, Sonora
***Asclepias fascicularis*** Decne.	Y	Y [34,40,58]		AZ: Pima [65,67]; CA: Inyo, Los Angeles, Riverside, San Bernardino, San Diego [68]; Mex: Baja CA
*Asclepias involucrata* Engelmann ex Torrey /*macrosperma*	Y [61]			AZ: Apache, Cochise, Coconino, Graham, Mohave, Navajo, Pima, Santa Cruz, Yavapai [58,61]; Mex
*Asclepias latifolia* (Torr.) Raf.	Y [6]			AZ: Apache, Cochise, Coconino Greenlee, Mohave, Navajo, Yavapai [58,61]; CA: Inyo [69,70], San Bernardino; UT [58]
*Asclepias linaria* Cavanilles	Y	Y [71,72]	Y	AZ: Cochise, Gila, Graham, Greenlee, Maricopa, Pima, Pinal, Santa Cruz [72], Yavapai [58,61,63,65,67,71]; Mex
***Asclepias nytaginifolia*** A. Gray	Y [58,61]	Y [24]		AZ: Apache, Cochise, Coconino, Gila, Graham, La Paz, Maricopa, Mohave, Navajo, Pima, Santa Cruz, Yavapai, Yuma [29,61,73,74]; CA: San Bernardino; NV [73]; Mex: Sonora, Baja CA
*Asclepias speciosa* Torrey	Y	Y [74]		AZ: Apache, Coconino, Gila, Greenlee, Navajo [61,63]; CA: Inyo [70,75], NV: Clark, Nye
***Asclepias subulata*** Decne.	Y [58,61]	Y [59,76,77]	Y [76]	AZ; Gila, Pinal, Maricopa, Mojave, Yuma [58,61] CA: Imperial, Riverside, San Bernardino, San Diego; CA [78]; NV; Mex: Baja CA, Sonora
*Asclepias vestita* Hook & Arn.	Y			CA: Inyo, Los Angeles [70], San Bernardino [79]
*Funastrum cynanchoides* (Decne.) Schltr.	Y [21,76]	Y [80]	Y	AZ: Cochise, Coconino, Graham, Pima, Pinal, Santa Cruz, Yavapai [61]; CA: Imperial, Orange, Riverside, San Bernardino, San Diego; Mex
*Funastrum cynanchoides* ssp *hartwegii* (Vail) R. Holm	Y	Y [80]		CA: Imperial, Riverside, San Bernardino, San Diego; UT; Mex
***Funastrum hirtellum*** **(A. Gray) Schltr.**	Y [61]	Y [29]		AZ: Coconino, La Paz, Mohave [61,67]; CA: Inyo, Imperial, Riverside, San Bernardino, San Diego; NV
*Funastrum utahense* (Engelm.) Liede & Meve	Y [61,77]	Y		AZ: La Paz, Mohave [67]; CA: Imperial, Riverside, San Bernardino, San Diego; NV

Y = yes, blank= unknown, *= not native; † = hypothesized nectar plant; bold type = indicates documented use by *D. g. thersippus*; + = contains PAs.

**Table 3:** Pyrrolizidine alkaloid (PA) host plant resource availability for *Danaus gilippus thersippus*. Pyrrolizidine alkaloid (PA) plant resource availability for *Danaus g. thersippus* in the Mojave and Sonoran Deserts in the US and Mexico, including larval host plants and nectar plants.

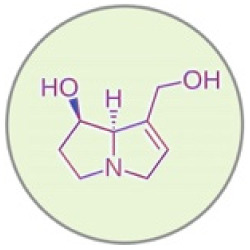 Nectar plant used by queen butterfly containing PAs						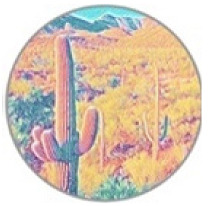 Found in desert	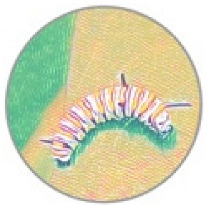 Larval host plant	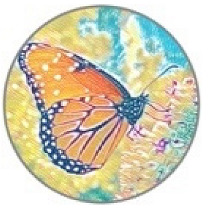 Nectar plant	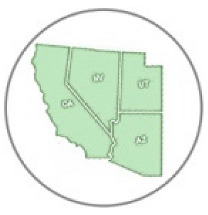 Distribution in Arizona (AZ), California (CA), Nevada (NV), and Utah (UT) and Mexico (Mex)
**Asteraceae**									
*Ageratina* [81,82] (*Eupatorium*) *herbacea* (S. Gray) R. M. King & H. Rob. PAs						Y		Y other spp [65]	AZ: Pima; CA: San Bernardino [83]
*Senecio*† [70,81,83,84] *faccidus* Less. Var. *douglasii* (D.C.) B. L. Turner & T. Barkley PAs						Y		Y [85], Figure S1	CA: Inyo, Orange, Riverside, San Bernardino, San Diego [86]; Mex: Baja Ca
*Senecio* † [70,81] *faccidus* var. monoensis (Greene) B. L. Turner & T. M. Barkley PAs						Y [77]		Y [86], Figure S1	AZ, CA: Inyo, Mono; Riverside, San Bernardino, San Diego [87]; Mex: Baja Ca
*Chromolaena odorata* (L.) R. M. King & H. Rob. [58,82,88] PAs								Y [65]	Mex [61]
**Boraginaceae**									
***Amsinkia*†** [70,81,85,89] ***tessellata* A. Gray** PAs						Y [80]		†	CA: Imperial, Inyo, Riverside, San Bernardino [77], San Diego
***Amsinkia*†** [70,81,85] ***tessellata*** ***A. Gray var. tessellata*** PAs						Y		†	AZ; CA: Imperial, Inyo, Riverside, San Bernardino [77], San Diego [45]; Mex: Baja Ca
*Cryptantha* [81] *angustifolia (Torr.) Greene* PAs						Y		†	AZ: La Paz, Maricopa, Mohave, Pima, Yuma [63]; CA; Imperial, Inyo, Riverside, San Bern.; NV: Clark, Nye
*Cryptantha* [81] nevadensis A. Nelson & P.B. Kenn. PAs						Y		†	AZ: Coconino, La Paz, Mohave, Maricopa, Pima, Yuma [63]; CA: Kern, Inyo, Imperial, Los Angeles, Riverside, San Bernardino, San Diego, Santa Barbara; NV: Clark, Nye, UT: Washington
*Cryptantha* [81] utahensis (A. Gray) Greene PAs						Y		†	AZ: Maricopa, Mohave [63]; CA: Inyo, Imperial, Riverside, San Bernardino; NV: Clark, Nye; UT: Washington
***Heliotropium*** [70,81,85] ***curassavicum* L. var. *oculatum* (A. Heller) I.M. Johnst. ex Tidestr.** PAs						Y		Y [9]	CA: Imperial, Inyo, Orange, Riverside, San Bernardino, San Diego [90]; NV; Mex [33]
***Helitropium spp.***						Y		Y †	CA: Riverside, San Bernardino, Inyo [90]
*Tournefortia* [81] *floribunda* PAs (related to *Heliotropium*)								Y	Mex: Mexicali [24]
**Apocynaceae**									
Nerium oleander * L. PAs Naturalized, * not native						Y		Y [29,58]	CA: Los Angeles, Orange, Riverside, San Bernardino [91], San Diego [91]
*Matelea parvifolia* (Torr.) Woodson [70] PAs (Rare)						Y		Y [70]	CA: Riverside, San Bernardino, San Diego [92]
**Euphorbiaceae**									
*Croton*† *californicus* [93] Müll. Arg. Possibly glutarimide alkaloids & sesquiterpene guaiane-type alkaloids with antibiotic properties [94]						Y		Y [70] †	CA: Inyo, Kern, Los Angeles, Orange, Riverside, San Bernardino, San Diego [95]
**Lamiaceae**					
*Condea emoryi* (Torr.) Harley & J.F. B. Pastore Triterpenoids, butelinic acid which have anti-tumor, anti-inflammatory, antimalarial properties [60]						Y [18]		Y [18,38]	AZ [67]; CA: Imperial, Riverside, San Bernardino, San Diego [95]; Mex: Ensenada, Mexicali

+ = contains PAs; *= not native; † = hypothesized nectar plant; **bold type =** indicates documented use by *D. g. thersippus.*

**Table 4:** Nectar host plant resource availability and long-distance movement of *Danaus gilippus thersippus*. Nectar plant resource availability for *Danaus g. thersippus* in the western desert regions of the Mojave and Sonoran Deserts in the US and Mexico, including larval host plants and nectar plants.

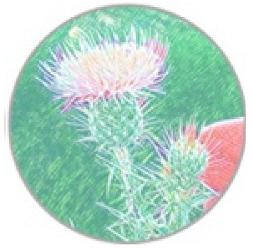 Nectar host plant used by queen butterfly	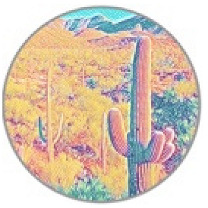 Found in desert	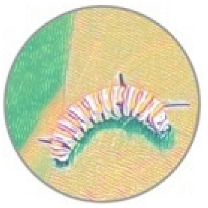 Larval host plant	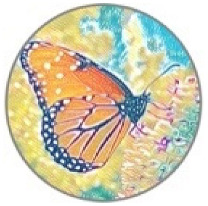 Nectar plant	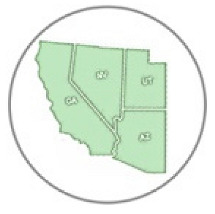 Distribution in Arizona (AZ), California (CA), Nevada (NV), Utah (UT) and Mexico (Mex)
**Asteraceae**				
*Acamptoppaus sphaeorcephalus* (Harv. & A. Gray) A. Gray	Y		Y [57]	AZ: Coconino, Gila, Graham, La Paz, Maricopa, Mohave, Pima, Pinal, Yavapai [67]; CA: Imperial, Inyo, Kern, Los Angeles, Riverside, San Bernardino, San Diego [95]; NV; Clark, Lincoln; UT: Washington, Kane, San Juan
*Baileya multiradiata* [96] Harv. & A. Gray ex Torr.	Y		Y [96]	AZ: Pima; CA: Inyo, Los Angeles, Orange, Riverside, San Bernardino, San Diego [95]
*Cirsium mohavensis* (Greene) Petr.	Y [97]		Y [70,98]	AZ 41; CA: Inyo [70,97,98]
***Heliomeris longifolia*** [Figure 3] (Robbins. & Greenm.) Cockerell	Y [57]		Y (Figure 3)	AZ: Cochise, Pima [57], Santa Cruz; NV, UT, Mex: central
***Palafoxia arida*** B. L. Turner & M. I. Morris	Y [32]		Y [32]	CA: Imperial Inyo, Riverside, San Bernardino, San Diego; Mex: Sonora [32]
*Ericameria (Chrysothamnus) nauseosa* (Pursh) G. L. Nesom & G. I. Baird var *mohavensis*	Y		Y	CA: Inyo, Los Angeles, Orange, Riverside, San Bernardino [37], [99]
*Helianthus annuus L.*	Y [100]		Y (Figure 3)	CA: Imperial, Inyo, Los Angeles, Orange, Riverside, San Diego [99]
Verbesina encelioides * (Cav.) Benth, & Hook f. ex A. Gray * Not native	Y		Y	CA: Orange, Los Angeles, Riverside, San Bernardino, San Diego
***Senecio flaccidus*** Less. **monoensis or douglasii** † PAs	Y † [42,100]		Y † [27]	CA: Inyo, Kern, Los Angeles, Riverside, San Bernardino, San Diego [86,87]
***Xanthisma spinulosum*** (Pursh) D. R. Morgan & R. L. Hartm.	Y [100]		Y [57]	AZ [100]; CA: Riverside, San Bernardino [45], NV; UT; Mex
**Lamiaceae**				
*Condea emoryi* (Torr.) Harley & J.F. B. Pastore	Y [77]		Y [18]	AZ; CA: Imperial, Riverside, San Bernardino, San Diego [100]; Mex: Ensenada, Mexicali [99]
**Fabaceae**				
*Parkinsonia florida* (A. Gray) S. Watson	Y		Y	AZ [25]; CA: Imperial [101,102], Riverside, San Bernardino, San Diego [39]; Mex: northwest
**Verbenaceae**				
*Phyla lanceolata* (Michx.) Greene	Y		Y [24]	CA: Inyo, Los Angeles, Orange, San Bernardino, San Diego
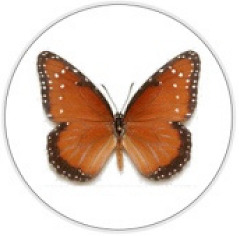 Queen movement	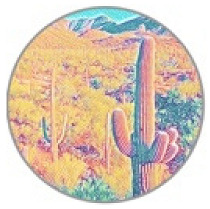 Found in desert	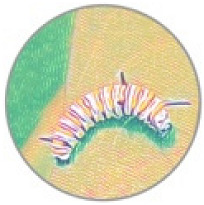 Larval host plant	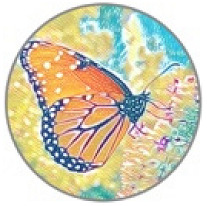 Nectar plant	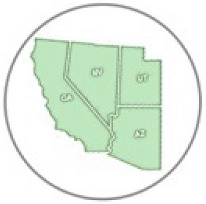 Distribution in Arizona (AZ), California (CA), Nevada (NV), Utah (UT) and Mex
*Danaus gilippus thersippus* Long distance movement documented by tagging [S2] 1. AZ to CA: **598.85 km** 2. AZ to CA: **671.42 km** 3. AZ to CA: **1404.31 km**	Y		See above	AZ: Cochise, Coconino, Maricopa, Mohave, Pima, Santa Cruz (SI) [67]; NV: Clark, Nye; CA: Imperial, Orange, Riverside, San Diego, San Bernardino; UT: Washington [S-2]; Mex: (SI) Baja Ca

+ = contains PAs; *= not native; † = hypothesized nectar plant; **bold type =** indicates documented use by *Danaus g. thersippus.*

## Supplementary Materials

The following are available online at https://www.mdpi.com/2075-4450/11/5/315/s1, Figure S1: *Danaus gilippus thersippus* nectaring on *Senecio flaccidus* host plant, Figure S2: Phenology of *Danaus gilippus thersippus* larval and adult host plants; Table S1: Data from GBIF, link and list of institutions, Table S2: Examples of *Danaus gilippus thersippus* specimens from museums with useful data on movement and host plant use.
