# Supplementary material for: Ecology of the Western Queen Butterfly Danaus gilippus thersippus (Lepidoptera: Nymphalidae) in the Mojave and Sonoran Deserts
Source: Insects. 2020 May 19;11(5):315. doi: 10.3390/insects11050315 (PMC7290759; doi:10.3390/insects11050315)
Supplement: Supplementary file 1 [file insects-11-00315-s001.zip › Supplemental Information_14_May_Danuas_gilippus_thersippus_Saul.docx]

**Supplemental Information**

Table S-1: Data from GBIF, link and list of institutions ([GBIF.org](file:///Users/lesliesaul/Desktop/queen%20paper%20&%20images/GBIF.org)) <https://doi.org/10.15468/dl.eca0s1> (Accessed 6 December 2019)

| Institutions on GBIF with datasets containing *Danaus gilippus thersippus* |
| --- |

1. California Academy of Sciences -Entomology Museum (CAS)
2. Computarización y actualización de la curación de la Colección de Lepidóptera del Museo de Zoología “Alfonso L. Herrera” y su base de datos MARIPOSA.FASE I
3. Cleveland Museum of Natural History (CMNH)
4. Abejas de México/Apoidea (CNIN)
5. Denver Museum of Natural History (DMNH)
6. Essig Museum of Entomology Collection UC Berkeley (EMEC)
7. Instituto de Biología, Universidad Nacional Autónoma de Mexico (IBUNAM)
8. iNaturalist Research-grade Observations
9. Natural History Museum of the Los Angeles County Museum (NHM LACM)
10. MZ Facultad de Ciencias (MCFC)
11. RR Holland collection
12. San Diego Natural History Museum (SDNHM)
13. Comisión Nacional para el Conocimiento y Uso de la Biodiversidad (CONABIO)

Additional resources with records

1. The Lepidopterists' Society Season Summary website *Danaus gilippus thersippus* <https://scan-bugs.org/portal/collections/listtabledisplay.php> p 314-316
2. Florida Museum of Natural History [http://www.flmnh.ufl.edu/lepsoc/)
3. Natural History Museum of Utah
4. BugGuide
5. Butterflies and Moths of North America
6. PollardBase-Orange County Butterfly Monitoring Network
7. Yale University Peabody Museum
8. Utah Museum of Natural History
9. BISON
10. Museum of Comparative Zoology, Harvard University
11. Natural History Museum (London)
12. Sam Noble Oklahoma Museum of Natural History
13. Botanic Garden and Botanical Museum Berlin-Dahlem Observationa

Table S-2. Examples of *Danaus gilippus thersippus* specimens from museums with useful data on movement and host plant use [The Lepidopterists' Society Season Summary website *Danaus gilippus thersippus* <https://scan-bugs.org/portal/collections/listtabledisplay.php> and [<http://www.flmnh.ufl.edu/lepsoc/>).

| Notes | Catalog # | Date | Locality | Locality | Record Id |
| --- | --- | --- | --- | --- | --- |
| 1. Location right on the Pacific Ocean Coast | LEPSOC_A_00104798 | 2016-ix-07 | USA, CA, Orange, San Clemente | 33.426973,  -117.611992  +-6787 m | 97e413dc-c4cb-4bf7-a41e-28c30f1bffa1 |
| 2. Individual tagged in Willcox, AZ on ix-20-2009 found in Joshua Tree NP, traveled 598.85 km | LEPSOC_A_00104800 | 2016-x-09 | USA, CA, Riverside, Joshua Tree NP  ***Travelled west from AZ to CA desert** | 34.134728,  -116.313066  +-3901 m | 43d399d8-d75d-4a00-a1f2-2e53efdd8951 |
| 3. ova on *Cynanchum [now Funastrum) utahense*,  host record | LEPSOC_A_00051370  https://scan-bugs.org/portal/collections/list.php?db=148;&page=1 | 1997-x-20 | USA, CA, Riverside, east end of Eagle Mtns. near Pinto Well | 38.925737,  -123.415842  +-301 m | d5a4a200-032e-4181-b7c6-696553eca768 |
| 4. ova on *Cynanchum [now Funastrum) utahense*, HOST RECORD;  J. Emmel & G. Pratt  originally submitted as *D. gilippus strigosus* | LEPSOC_A_00051369  *Danaus gilippus thersippus* | 1997-x-20 | USA, CA, Riverside, east end of Eagle Mtns nr Pinto Well | 38.925737,  -123.415842  +-301 m  **incorrect GPS** | fb5be6b7-ff27-4bee-bf2a-ace5ba512df8 |
| 5. John F. Emmel  Desert scrub | LEPSOC_A_00056678 | 1995-xii-10 | USA, CA, Riverside, Hemet | 33.74752,  -116.971968  +-9054 m | 064751ef-4bd2-4caa-b6af-a8603d6c2d5c |
| 6. Hartmut Wisch  adult butterfly | BugGuide image | 2008-ix-18 | USA, CA, Los Angeles, Santa Ana Botanic Garden |  | https://bugguide.net/node/view/226424 |
| 7. R. Hannawacker  Butterfly Count Group | LEPSOC_A_00090509 | 2014-ix-06 | USA, AZ, Coconino, Grand Canyon NP: Phantom Ranch | 36.104983,  -112.095169  +-3036 m | 6cf6c71f-f1d8-480d-a6ad-935f5eb1a8b1 |
| 8. not overwintering in Mexico or the Pacific Coast  desert | LEPSOC_A_00041660 | 2000-xii-11 | USA, AZ, Mohave, Havasu Springs, nr. Parker | 36.216649  -112.686857  +-90 m | b3d8b503-83c4-4641-8806-a31bd0e681f6 |
| 9. A. Grkovich  desert | LEPSOC_A_00041658 | 2000-xii-11 | USA, AZ, Mohave, Craggy Wash Canyon nr. Lake Havasu City & vicinity | 34.58443,  -114.368713  +-11627 m | 6d3865e1-1f94-4024-ae5d-a7042b1d70e9 |
| 10. G. Forbes  'mesquite wash'  Denver Mus. of Sci. | ZE.3494 | 1972-ix-08 | USA, AZ, Maricopa, '10 mi. S. Sunflower' | 33.74314  -111.465417  +3881 m  WGS84 | d03e0f35-f727-40dc-baf2-f537a25c2c04 |
| 11. Patrick Dockens | BugGuide | 2005-iv-03 | USA, AZ, Maricopa, Peoria along New River |  | https://bugguide.net/node/view/41827 |
| 12. Same tagged individual seen at Joshua Tree NP  X-9-2016,  traveled 671.42 km | LEPSOC_A_00104642 | 2016-ix-20 | USA, AZ, Cochise, Willcox, Muleshoe Ranch [Nature Conservancy)  **•traveled west** | 32.338962,  -110.237021  +-3036 m | d439fee7-9cde-4ea9-90f3-9f52e5095dc4 |
| 13. Jessica Griffiths Recovered at Pacific Grove, Monterey Co., CA 26-x-14, traveled 1404.31 km | LEPSOC_A_00090532 | 2014-ix-22 | USA, AZ, Santa Cruz, Canelo Forest Administrative Area    ***Traveled west from AZ to CA coast**. | 31.542872.  -110.514526  +-3036 m | 5f2cbbee-3d20-4f0e-891f-2e8d7c3fdb7d |
| 14.Bryan  Brasmussen | Image iNaturalist | 2017-vi-20 | Baja, CA | 30.9674111111,  -115.74174444  44 |  |
| 15. R.C. Salisbury  Natural History Museum of Utah | UMNH.ent.0052341 | 1953-iii-22 | USA, CA, San Diego, Borrego Desert State Park | 33.133795,  -116.301218 +21723 m, WGS84 | f66a048a-2f04-4af1-9068-58bb87e3758c |
| 16. Kenneth B. Tidwell  Nat. Hist. Mus. Of Utah | UMNH.ent.0071652 | 1984-x-03 | USA, AZ Pima, Why | 32.210594  -112.757985  +560 m  WGS84 | dda5812d-55c2-4c2b-95df-141a4806d6a2 |
| 17. LATE DATE for northern desert | LEPSOC_A_00038569 | 2001-xi-10 | USA, CA, Kern, Ridgecrest | 35.622456,  -117.670897  +-8750 m | cd557088-035b-4a00-a0a9-7336386ddbc3 |
| 18. | LEPSOC_A_00090508 | 2014-ix-16 | USA, CA, Tulare, Kern River south of Corral Creek | 35.850804.   -118.452421  +-210 m | 0c338c78-5659-4603-a30f-b8488d9e63dc |
| 19. Photo by Melina Staal, first record in 28 years for this county | LEPSOC_A_00104797 | 2016-vii-31 | USA, CA, Mariposa, Mariposa, 4100 Triangle Rd | 37.484938,   -119.966284  +-3438 m | c1e40b19-6580-4df4-a019-ddb43c0cdeb8 |
| 20. | LEPSOC_A_00021592 | 2006-viii-23 | Mex, HIDALGO, Huautla, El Aguacate | 20.383333,  -98.166667  +-3036 m | c5f1cbda-0248-414b-8ae2-f25fc1f8e9c8 |
| 21. | LEPSOC_A_00111832 | 2015-iv-22 | Mex, Zacualpan, Parque Estatal _??Picacho de Oro y Plata_ѝ | 31.367055  -115.653643  +-301 m | b56bafa3-7569-4d14-98d1-4efabb366b84 |
| *N*=21 records |  |  |  |  |  |


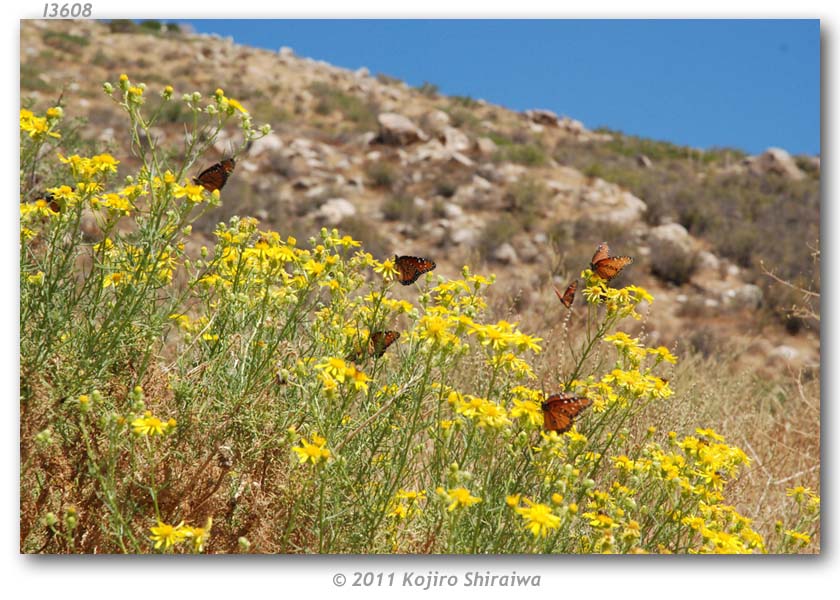


**Figure S-1**. *Danaus gilippus thersippus* nectaring on *Senecio flaccidus* host plant. *Danaus gilippus thersippus* in San Felipe Hills, San Diego Co., CA, USA, 24 September 2008 (© Kojira Shiraiwa 2011, all rights reserved) on *Senecio flaccidus* (var. *douglasii* or *monoensis*) (J. Andre, per. com. 2019).

**Figure S-2.** Phenology of *Danaus gilippus thersippus* larval and adult host plants. Phenology of *D. g. thersippus* larval and adult host plants in the western Mojave and Sonoran deserts.
